# Dendrogramic Representation of Data: CHSH Violation vs. Nonergodicity

**DOI:** 10.3390/e23080971

**Published:** 2021-07-28

**Authors:** Oded Shor, Felix Benninger, Andrei Khrennikov

**Affiliations:** 1 Felsenstein Medical Research Center, Beilinson Hospital, Petach Tikva 4941492, Israel; shor.oded@gmail.com (O.S.); felixbenninger@gmail.com (F.B.); 2 Sackler Faculty of Medicine, Tel Aviv University, Tel Aviv 6997801, Israel; 3 Department of Neurology, Rabin Medical Center, Petach Tikva 4941492, Israel; 4 Department of Mathematics, Faculty of Technology, Linnaeus University, 35195 Växjö, Sweden

**Keywords:** ontic, epistemic, dendrograms, dendrogramic holographic theory, clustering algorithms, quantumness, CHSH inequality, nonergodicity

## Abstract

This paper is devoted to the foundational problems of *dendrogramic holographic theory* (DH theory). We used the ontic–epistemic (implicate–explicate order) methodology. The epistemic counterpart is based on the representation of data by dendrograms constructed with hierarchic clustering algorithms. The ontic universe is described as a p-adic tree; it is zero-dimensional, totally disconnected, disordered, and bounded (in p-adic ultrametric spaces). Classical–quantum interrelations lose their sharpness; generally, simple dendrograms are “more quantum” than complex ones. We used the CHSH inequality as a measure of quantum-likeness. We demonstrate that it can be violated by classical experimental data represented by dendrograms. The seed of this violation is neither nonlocality nor a rejection of realism, but the nonergodicity of dendrogramic time series. Generally, the violation of ergodicity is one of the basic features of DH theory. The dendrogramic representation leads to the local realistic model that violates the CHSH inequality. We also considered DH theory for Minkowski geometry and monitored the dependence of CHSH violation and nonergodicity on geometry, as well as a Lorentz transformation of data.

## 1. Introduction

A model for the Universe based on the hierarchic relational representation of its components was suggested in [1]. The present paper is devoted to the foundational problems structured within Primas and Atmanspacher’s [2,3] ontic–epistemic and Herz and Boltzmann’s [4,5] descriptive observational structuring of physical theories (see also [6,7,8]), as well as Bohm’s [9] implicate–explicate order viewpoint on the Universe. One of the aims of our study was to re-establish local realism in physics (cf. [10,11,12,13,14,15,16,17,18]). 

We describe the representation of experimental data by dendrograms using *hierarchic clustering algorithms*. They lead to the p-adic model at the ontic level. (From this viewpoint, our research is part of p-adic theoretical physics, see, for example, [19,20,21,22,23,24,25,26,27,28] and Section 6.) In [1], we named our theory *dendrogramic holographic theory* (DH theory). In this paper, we do not discuss holography (see [1]) but proceed with the same name. 

*The* p-adic *tree* (*p* > 1 is a natural number) is the infinite tree with a homogeneous structure of branching at each node, one incoming and p-outcoming edges. This tree is endowed with a natural metric (so-called “ultrametric”) and the algebraic structure of a ring (see Appendix A). 

We recall that an ontic theory is about reality as it is, and an epistemic theory is about knowledge collected through observations. The ontic universe is unapproachable by observers. Nevertheless, its structure can be theoretically reconstructed by increasing the size of dendrograms; observer *O* generates approximations of the ontic universe. The limiting mathematical structure is uniquely determined by the p-adic tree (or algebraically, the p-adic ring, denoted as **Zp**). 

One of the main aims of novel mathematical modeling of ontic–epistemic structuring is to better understand the classical–quantum interrelation. Recalling that *quantum mechanics is an epistemic model*, it is about observations’ outcomes (see Bohr [29,30] and Plotnitsky [31,32,33]). In DH theory, “quantumness” is present only at the level of dendrograms, and the p-adic ontic universe is classical. The heuristic criterium of quantum-likeness is very natural: simple dendrograms are more quantum(-like) than complex dendrograms. As a test of quantum-likeness, we used the *CHSH inequality*, not for the standard observables, but for new observables reflecting the hierarchic relational structure of data. It should be highlighted that the original experimental data used for this paper are classical. Its quantum-like structure becomes visible through dendrogramic representation. The seed of the violation of the CHSH inequality is neither a nonlocality nor a rejection of realism, but the *nonergodicity* of dendrogramic time series. The dendrogramic representation leads to a local realistic model that violates the CHSH inequality.

This is a good place to recall that a few authors have coupled quantum behavior with the violation of ergodicity [34,35,36,37,38]. We also considered DH theory for Minkowski geometry and monitored the dependence of CHSH violation and nonergodicity on geometry as well as a Lorentz transformation of data.

The reader who is not interested in foundations can jump directly to Section 7, which is devoted to the experimental data, correlations, and the violation of the CHSH inequality for hierarchic observables and its correlation with the nonergodicity of data. 

## 2. Dendrogram Representation of the Universe

### 2.1. Systems as Clusters of Clicks of Detectors

According to Bohr, the outcomes of measurements are not the objective properties of systems [29,30,31,32,33]. They quantitively represent interrelation between a system *S* and an observer *O*. An ontic system exists independently of *O*. However, it is unapproachable by the observer; in fact, *O* constructs its approximate representation by using data. 

Consider an observer *O* constructing a representation of a surrounding environment—in an extreme case, of the Universe. In DH theory, the observer has the free choice not only to perform or not perform some observation, but even the free choice to decompose the collected data into blocks and to treat these blocks as the (epistemic) systems’ representation. *The fundamental entities of the epistemic theory are events* (cf. [14,39,40,41,42,43]), *not systems.* Systems are composed of a certain number of events (clusters of events), for example, several clicks of detectors (or other detection events). This position is very close to the view of Zeilinger on quantum mechanics (learned through private discussions with Akh).

### 2.2. Representation of Systems by Dendrograms

A simple collection of experimental statistical data does not tell observer *O* about the *genuine hierarchic* interrelations between physical processes under observations. Such hierarchic structure can be approximately recovered with the aid of *clustering algorithms.* This hierarchic clustering leads to the treelike geometry. In DH theory, (epistemic) systems are described by *dendrograms*, or finite trees. Any observer *O* can reconstruct their environment and, in the limit, the whole *O-*Universe, by collecting observational data. 

**Figure 1:**
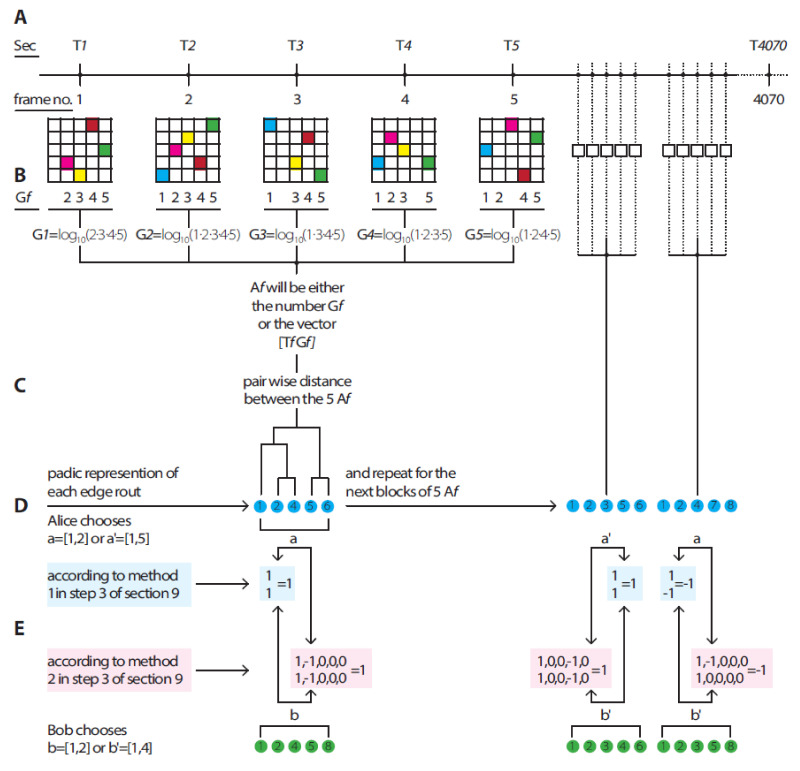
Abstract and numerical examples of the process from real data of a CCD chip (illustrated as a 5 × 5 CCD chip). Double-slit experiment via the construction of a dendrogram (with 5 edges corresponding to 5 frames) with a series of 1 and −1 values, from which CHSH correlations were calculated.

In DH theory, the *decomposition of the O-Universe into subsystems is done through the decomposition of data into blocks*. Each block of data is represented by a dendrogram—this is a system. The data can be subdivided into blocks in different ways. The *O*-Universe can be decomposed into systems of different complexities depending on the block’s size. We illustrate this process in Figure 1 by dividing data into blocks of equal size, where d = 5.

### 2.3. p-Adic Metric for Hierarchic Relations

The points at the baselevel of any dendrogram *D* (see Figure 1) represent the relational characteristics of the system expressed by *D*. For the simplest hierarchic algorithms based on the choices of just two alternatives—two branchings at the vertexes, the base points can be described by vectors with coordinates 0/1. The natural distance between these characteristics is given by 2-adic ultrametric *r_2*; the longer the common root of the paths going from the dendrogram’s root to points *a*,*b*, the shorter the distance between them (see Appendix A). This metric encodes the hierarchic relations inside the dendrogramic system and with its surrounding environment. More general hierarchic clustering algorithms with *p*-branching at vertexes generate the representation of systems by dendrograms having more complex topology. (Here, *p* > 1 is a natural number determining the tree structure). The branches (and the corresponding endpoints) are encoded by vectors with the coordinates belonging to the set {0, 1, …, *p* − 1}. The corresponding common root distance is denoted by *r_p.*

Take two points, *a*,*b*, at the baselevel of dendrogram *D* representing a system. The common root distance *r_p(a,b)* gives the quantification of the closeness of these two systems’ characteristics with respect to the hierarchic order relation. The quantity

*R_D = max{r_p(a,b): a, b belong to D}*

(dendrogram’s diameter) determines the degree of hierarchic interrelation between the elements of the dendrogram system. If *R_D* is small, the system’s characteristics are highly relationally connected, if *R_D* is large, the system is relationally sparse. 

### 2.4. Epistemic Realism, Hidden Variables

Each dendrogram *D* can be represented by the vectors with coordinates belonging to the set {0, 1, …, *p* − 1}. These are (hierarchic relational) *hidden variables*. Observer *O* can invent relational observables defined as functions of these hidden variables, for example, A = A(u). This is *the realistic model of observations*, which is constructed by the observer *O* through hierarchic structuring of the experimental data. This approach can be called *realism without reality*, as opposed to the *reality without realism* viewpoint on quantum physics suggested by Plotnitsky [33,44]. Such hidden variables are constructed on the basis of experimental data; they are, so to say, epistemic variables. However, the mathematical model is realistic in the sense of a set-theoretical representation of states as function of hidden variables and observables. 

## 3. From Epistemic (Explicate) to Ontic (Implicate): p-Adic Universe

By considering the data blocks of increasing size, in the limit, *O* can reconstruct the ontic description. The dendrogram-based epistemic theory leads to the p-adic geometry of the ontic universe. (The role of the model parameter p is discussed in Section 5.) In the limit, dendrograms of increasing size generate the infinite p-adic tree endowed with the p-adic ultrametric. Its infinite branches are points of the ring ***Z_p***; in fact, ***Z_p*** coincides with the unit ball. Thus, with respect to the hierarchic p-metric, *the Universe is bounded.* Topologically the p-adic universe differs crucially from the Universe endowed with the Euclidean or Minkowski geometry. The basic properties of ***Z_p*** match very well with Bohm’s views on implicate order. This space *is disordered, zero-dimensional, and totally disconnected.*

## 4. From Ontic (Implicate) to Epistemic (Explicate)

In Section 3, the mathematical structure of the ontic universe was approached from the special dendrogram representation of experimental data. Now, we proceed with another way around—from the ontic p-adic model to the epistemic dendrogramic model. Our previous epistemic-to-ontic pathway (Section 3) centralized the role of an observer *O*. This can make the impression that the personal observer perspective plays a crucial role in DH theory (cf. with QBism [45]). Now, by starting with the p-adic ontic model, we diminish the subjective component.

The points of the p-adic tree ***Z_p*** represent all possible events that can happen in the Universe. Thus, as well as the epistemic universe, the ontic universe is represented as a set of events. However, these are not observational events; they cannot be associated with, say, the clicks of detectors. We consider the p-adic points *absolute events*. An absolute event is the endpoint of the infinitely long path of the p-adic tree; mathematically, the sequence is

*a = a*1*….an …., with coordinates aj = *0*, *1*, …, p − *1*.*

Each finite cutoff of the infinite path *a* is an element of the explicate order, and it can be interpreted as the characteristic of a physical system. The latter is determined in a variety of ways depending on the embedding of the finite path *a’ = a*1*….an* into various dendrograms. We considered the following process of the structuring of the ***Z_p*** universe. Let us select some set of points *S* of ***Z_p*** (finite or infinite). These points are geometrically presented as paths starting at the root of the p-adic tree. Thus, *S* is a subtree of this tree. The common roots of these paths determine the relational closeness of corresponding points in *S*. As well as in the epistemic model, these points can be considered as characteristics of *S.* Observer *O* is not involved in this consideration. Roughly speaking, the system *S* exists irrespectively, whether somebody looks at it or not. 

Now, take the tree of an ontic system *S* and cut it at some level, say, *n* steps from the root. Such a dendrogram expresses a system *S_n* belonging to the explicate level of nature’s description. In principle, there is no need to couple it to some observer. This construction provides the realist interpretation, even for systems represented by finite dendrograms. The only subjective element is the place of cutoff (cf. with the problem of the boundary between the quantum and classical worlds). 

## 5. The Role of the Clustering Algorithm

Different hierarchic clustering algorithms generate different dendrograms and observers’ free will also covers the algorithm’s choice. However, in the ontic description, this dependence on the algorithm’s choice is washed out. All algorithms with p-alternative branching generate approximations for the same ontic model of the Universe, given by the p-adic tree. The rings of p-adic numbers ***Z_p*** are not isomorphic for a different *p*. However, their topological and algebraic properties are the same. Similar problems have been discussed in p-adic theoretical physics [19,20,21,22,23,24,25,26,27,28]: What *p* does correspond to the physical reality? There are various suggestions, including such exotic choices as *p* = 127 [26]. The pragmatic solution was to treat all p-adic models on equal grounds and consider the p-adic encoding similarly to the representation of real numbers by using different bases.

Of course, there exist clustering algorithms that generate nonhomogeneous trees, with different branching indexes for different vertexes. Such dendrogramic models lead to arbitrary trees (arbitrary ultrametric spaces). One may try to exclude such models by appealing to the relational scale homogeneity of nature. In any event, we keep to p-adic models. Our original choice of the concrete hierarchic clustering algorithm [1] was motivated by studies, including the data analysis of Murtagh [46,47,48]. In the present study, we played with a few other algorithms, but their outputs are very similar with slight differences in details. In Section 11.3, we apply seven different linkage clustering algorithms with no apparent differences in CHSH value outcomes (Figure 5 in Section 11.3). We refer the interested reader to the Matlab software website for details on those seven linkage clustering algorithms [49]. 

## 6. “Traditional p-Adic Theoretical Physics”: Emphasis of the Number-Theoretic Structure

On p-adic trees, it is possible to introduce arithmetic operations; algebraically, these spaces are similar to the real line. However, topologically p-adic trees differ crucially from the real line. p-adic theoretical physics (see, e.g., [19,20,21,22,23,24,25,26,27,28] and references therein) flourished in the 1990s, stimulated by string theory [19,21,22]. Its main problem, which finally led to an essential dampening of its development during recent years, was the absence of coupling with real experimental data. Roughly speaking, there are no measurement devices that generate p-adic outputs. However, as was clearly stated by Volovich [26], there are also no measurement devices that generate irrational numbers; due to the finite precision of measurements, all their outputs are rational numbers. Volovich claimed that the only physical numbers are rational numbers. Since rational numbers are dense, both in real and p-adic fields, Volovich suggested the unification of standard and p-adic physics on the basis of rational numbers. Although he did not use the ontic–epistemic terminology, we can say that, for him, mathematically, epistemology is based on rational numbers. Beyond this general rational epistemology, one can recover the real and p-adic ontic models. We suggest another epistemology for p-adic ontology. This is the hierarchic relational epistemology. It differs crucially from the real-order epistemology of “standard physics”. We can say that Volovich’s approach was number-theoretical and our approach is hierarchic relational. 

The DH framework provides the rigid coupling of theory and experimental data. We hope that it will lead to the renaissance of p-adic modeling in physics. (We guess that its main theoretical results can be adapted to DH theory.)

## 7. Dendrogram Viewpoint on Classical–Quantum Interrelation

In our epistemic model, the sharp classical–quantum separation disappears. *The degree of classicality is based on a system’s complexity—*the size and topological complexity of its dendrogram representation. Quantum systems are characterized by a low complexity of their dendrograms. Thus, electrons and atoms can behave as quantum in some measurements because they have a very simple hierarchic structure of interrelation between their components. In our approach, not only can systems be described by quantum mechanics but even classical physical systems can exhibit quantum(-like) behavior within a hierarchic representation of experimental data.

Bell-type inequalities (and in particular, the CHSH inequality) have been used to show the “quantumness” degree of photons or particle systems [50,51,52,53,54,55]. We selected four system characteristics (the endpoints of dendrograms representing systems) *a,a’, b,b’* and calculated the correlations for pairs *(a,b), (a,b’), (a’,b)*, and *(a’,b’).* Finally, a CHSH combination of correlations was formed and the problem of its exceeding of two was studied via a numerical simulation. For Alice and Bob, the fixed settings are *a,a’* and *b,b’*, respectively. The experimenter calculated the correlations Cab, Cab’, Ca’b, Ca’b’, and then the CHSH combination of them, as follows:

C = Cab − Cab’ + Ca’b + Ca’b’

*Inequality |C| > 2 means data collected in experimental runs is consistent with predictions of quantum theory and contradicts local realism, that is, it cannot be described by a hidden variable model that is local.*

We considered two time series of experimental data. The data was collected in measurements of one fixed classical physical observable. The outcomes of this observable carry the information about hierarchic relations between the characteristics of a physical system under measurement. (The data was decomposed in the systems by the observer.) These relations are not visible in the straightforward graphic representation of the data (see Figure 1). They were extracted from “real data” with the aid of a clustering algorithm.

The standard viewpoint on the violation of the Bell-type inequalities [50,51,52,53,54,55] is that it shows that *“quantum correlations are stronger than classical ones.”*

By trying to explain this difference between quantum and classical correlations, Bell and his followers pointed to a contradiction between quantum theory and local realism. The latter is mathematically formalized as a representation of observables by functions of hidden variables; locality means that, say, Alice’s functions do not depend on the settings selected for Bob’s functions, that is, Sa = Sa (u), where u is the hidden variable.

The interpretation of the violation of the Bell-type inequalities is the subject of hot foundational debates (see, e.g., [56,57,58,59,60]). Plenty of so-called loopholes were pointed out. Two basic ones, the nonlocality and the detection efficiency loopholes [61,62], were successfully closed in 2015 experiments [63,64,65]. 

## 8. The Role of Nonergodicity

De Broglie [66] was the first to highlight that experimentally obtained probabilities may deviate from the ontic probability distribution of hidden variables. He justified this possibility within his double solution theory in terms of the pilot wave. It seems that Krennikov’s works [67,68,69] were the first publications in which the difference between the frequency experimental probabilities and the measure-theoretic distributions of hidden variables was pointed out. Khrennikov appealed to the von Mises frequency probability theory. The latter straightforwardly implied that the Bell-type inequalities can be violated for local models. However, it seems to be impossible to mathematically formalize the notion of realism within the von Mises frequency theory [70]. Finally, in [37], the interplay between frequency and measure-theoretic probabilities was structured within the *ergodic theory* and it was shown that the violation of ergodicity is a sufficient condition for the violation of the Bell-type inequalities. However, this was shown in a purely theoretical framework. In our further considerations, we show that in DH theory, the Bell-type inequalities are violated because of the nonergodicity of the time series of experimental data restructured with hierarchic clustering algorithms.

Hence, in DH theory, the Bell-type inequalities are not a tool for the rejection of the local realistic description or the classical probability model (Kolmogorov [71], 1933). We use these inequalities to express the quantumness degree of experimental statistical data—in the spirit of the article by [72]. 

As was mentioned in Section 1, a few authors have pointed out that quantum effects can be derived in a classical statistical framework by rejecting the assumption of ergodicity [34,35,36,37,38]. In particular, in [35], it was guessed that the originally very emphasized the difference between quantum and classical statistical modeling can be eliminated by rejecting the hypothesis of the ergodicity of data; even black body radiation can be modeled classically, but within nonergodic statistical mechanics. In the paper by [37], *nonergodicity was pointed as one of the sufficient conditions for the violation of the Bell-type inequalities*, a condition that is equally important as nonlocality and a rejection of realism (the hidden variable description). 

DH theory provides the possibility of introducing hidden variables (of the special type) beyond any experimental data and considering a class of realistic observables, those represented as functions of hidden variables. Hence, straightforwardly, it seems that Bell-type inequalities cannot be violated under the assumption of locality. However, we show the violation of the CHSH inequality for special selection of “settings” that determine the observables. The reason for this is precisely the violation of ergodicity; the measure-theoretic and frequency averages do not coincide [37].

However, the natural question arises: Why do the hierarchically determined observables violate the assumption of ergodicity? The answer to this question is that the hierarchical sub-systems have already encoded in them the hierarchical structure of the “universal” dendrogram, as was demonstrated in our paper [1]. 

We directly check the hypothesis of ergodicity in Section 10 and we show that for a wide range of “settings”, the hierarchic observables are not ergodic. However, we also found that for some “settings”, the observables are ergodic, but, nevertheless, the CHSH inequality is violated. How does this happen? This situation was also discussed in paper [37]: 

“Consider two stationary ergodic processes *x*(*t*) and *y*(*t*). Is the vector process *z*(*t*) = (*x*(*t*), *y*(*t*)) ergodic? The answer is “no”. We remark that this implies that the product *x*(*t*)*y*(*t*) of two ergodic processes need not be ergodic. Thus, in principle, in quantum theory nonergodicity can be generated by measurements on compound systems. At the same time, measurements of each system separately generate ergodic stationary processes. In such a case, measurements on compound systems really have the special feature, nonergodicity.”

In addition, we really found that in the case of the violation of the CHSH inequality, the product of Alice and Bob observables is always nonergodic.

## 9. Scheme for the Calculation of Dendrogramic Correlations

For concreteness, we consider *p* = 2.

**Step** **1.** From a data time series to a dendrogram: a hierarchic clustering of data.

Consider a time series of some data, *Z*1*, Z*2*, …, Zn.* Split it into blocks of length *d.* (In Figure 1, these blocks have a length of 5). For each block, we constructed (with a hierarchic clustering algorithm) a dendrogram with *d* basepoints. We obtained a dendrogram time series, *D*_1_*, D_*2*_, …, D_s_*. All dendrograms have *k* levels, where 2*^k^*^−1^ *< d* ≤ 2*^k^.* (In Figure 1 step A, *d* = 5**, hence, *k* = 3.)

**Step** **2.** From a dendrogram time series to a 2-adic time series.

First, we remark that each dendrogram *D* can be represented by vectors of the form (*α*0*,α*1*, ..., αk*)**, where *αj* = 0**,1. Each vector encodes a path on *D* from its root to the corresponding endpoint; *D* is the selection of *d* vectors from 2*^k^* vectors.

From this vector representation, we moved to representations by natural numbers, which we define as features of the dendrogram, by using the following formula:

(*α*_0_*α*_1_*...α_k_*) = *α*_0_ + *α*_1_2 + *α*_2_2^2^ + *...* + *α_k_*2*^k^.*

Thus, from a series of dendrograms *D*_1_*, D_*2*_, ..., D_s_*, we obtained a series of natural numbers, which were sorted in ascending order in each block of length *d*, according to the hierarchical structure of each dendrogram *D_n_*. 

These natural numbers play the role of polarization in the real CHSH experiment. Later, we define new observables depending on such analogs of polarization. These d-blocks of natural numbers can be treated as hidden variables. New observables will be functions of these “hierarchic hidden variables”.

Then, for correlations, we considered two time series, *Z*1, *Z*2, …, *Zn* and *Z*1′, *Z*2′, *…, Zn’*. By applying the procedures of Steps 1 and 2, we obtained two series of dendrograms *D*_1_*, D_*2*_, ..., D_s_* and *D’*_1_*, D’*_2_, *..., D’_s_*, and two corresponding series of natural numbers. They were formed from consecutive blocks of *d* natural numbers, which were generated through the d-decomposition of the original time series: 

*x = x_*1*_, x_*2*_, ..., x_n_, x_n_ is composed of blocks with d natural numbers*

*y = y_*1*_, y_*2*_, ..., y_n_, y_n_ is composed of blocks with d natural numbers*

**Step** **3.Defining the observables.**

**First method** (see Figure 1 step E).

We constructed two time series of dendrograms, one for **Alice A = (A1, A2, A3, …, An)** and one for **Bob B = (B1, B2, B3, …, Bn)**.

For **Alice,** we selected two pairs of numbers, **a** = [a1 a2] and **a’** = [a1′ a2′]. The two pairs are not identical. The analogs of the two vectors are orientations of polarization beam splitters or Stern–Gerlach magnets.
For **Bob,** we selected two pairs of numbers, **b** = [b1 b2] and **b’** = [b1′ b2′]. The two pairs are not identical.
For each Ai i∊1, 2, 3, …, n, we randomly decided between pair a or a’.
For each Bi i∊1, 2, 3, …, n, we randomly decided between pair b or b’.
If, for **A**i, we chose pair **a,** we obtained the following:
If **A**i had both of the numbers in **a,** Sai = 1, otherwise, Sai= −1.

Metaphorically, we can say that if the “polarization” of **A**i coincides with a, the detector with the output is +1 clicks, if not, the detector with the output is −1 clicks.

We proceeded in the same way for the selection of a’ and b, b’. Then we calculated the correlations, as follows:

Cab = (∑Sai*Sbi)/length(a and b are selected together)
Cab’ = (∑Sai*Sb’i)/length(a and b’ are selected together)
Ca’b = (∑Sa’i*Sbi)/length(a’ and b are selected together)
Ca’b’ = (∑Sa’i*Sb’i)/length(a’ and b’ are selected together)
C= Cab − Cab’ + Ca’b + Ca’b’

**Second method** (see Figure 1 step E).

We constructed two time series of dendrograms, one for **Alice A = (A1, A2, A3, …, An)** and one for **Bob B = (B1, B2, B3, …, Bn)**.

For **Alice,** we selected two pairs of numbers, **a** = [a1 a2] and **a’** = [a1′ a2′]. The two pairs are not identical.
For **Bob** we select two pairs of numbers **b**=[b1 b2] **b’**=[b1’ b2’] the two pairs aren’t identical.
For each Ai i∊1,2,3…n, we randomly decided between pair a or a’
If for **A**i we chose pair **a** we have the following
We replace the natural number in Ai that equal a1 to 1
We replace the natural number in Ai that equal a2 to −1
We replace all other natural numbers in Ai to zero. We indicate that pair **a** was chosen to Ai.
The same we do for other settings, a’, b, b’-
We find i that a and b were chosen = nab,
We find i that a’ and b were chosen = na’b,
We find i that a and b’ were chosen = nab’,
We find i that a and b’ were chosen = na’b’,
We calculated Sab = (Ai = nab) *(Bi = nab).
We calculated Sa’b = (Ai = na’b) *(Bi = na’b).
We calculated Sab’ = (Ai = nab’) *(Bi = nab’).
We calculated Sa’b’ = (Ai = na’b’) *(Bi = na’b’).
For block j of size d of Sab, if there are two values of 1, Xabj = 1, if there is one value of 1, Xabj = 1, if there is one value of −1 Xabj = −1, if all values are 0, Xabj = −1.
For block j of size d of Sab, if there are two values of 1, Xa’bj = 1, if there is one value of 1, Xa’bj = 1, if there is one value of −1, Xa’bj = −1, if all values are 0, Xa’bj = −1.
For block j of size d of Sab, if there are two values of 1, Xab’j = 1, if there is one value of 1, Xab’j = 1, if there is one value of −1, Xab’j = −1, if all values are 0, Xab’j = −1.
For block j of size d of Sab, if there are two values of 1, Xabj = 1, if there is one value of 1, Xa’b’j = 1, if there is one value of −1, Xa’b’j = −1, if all values are 0, Xa’b’j = −1.

We calculated the following:

Cab = (∑Xabj)/length(Xabj)
Cab’ = (∑Xab’j)/length(Xab’j)
Ca’b = (∑ Xa’bj)/length(Xa’bj)
Ca’b’ = (∑ Xa’b’j)/length(Xa’b’j)
C = Cab-Cab’ + Ca’b + Ca’b’

## 10. Nonergodicity Check Methods

Nonergodicity means that measure-theoretic and frequency averages do not coincide. We claim that this is the main reason for the violation of the CHSH inequality. We have operated with the frequency averages. 

S_ab_ = <Sa,Sb>, but the standard proofs of the Bell-type inequalities were done for measure-theoretic correlation S_ab,measure_ = <<Sa,Sb>>. In order to check if our data was ergodic, we continued with the first scheme.

### 10.1. Scheme for Determining if Data Is Nonergodic

We started with one sequence *A* = (*A*1,*A*2, *..., An*), where each *d*-vector *Aj* = (*u*1*,u*2*, ..., ud*) represents a dendrogram. We fixed *a = [a*1* a*2*]* and defined

(1) $$Sa=Sa\left(u\right)$$

We continued to calculate *Sa as in the first method of Step 3 in Section 9*. We stress that (1) is the condition of realism, and the value of hidden variable *u* determines the outcome of the observable *Sa.* Thus,

(2) $$<Sa>=(\overset{n}{\underset{j=1}{\sum }}Sa\left(A_{j}\right))/N\;$$

is the frequency average. Next, we computed the probability distribution of hidden variables. We fixed one *d*-vector *u*, which is present in our dendrograms, and calculated the proportional number of occurrences of *u*, as follows.

(3) $$p\left(u\right)=\frac{number\;of\;occurrences\;of\;u}{n}$$

Then

(4) $$\ll Sa\gg =\underset{u}{\sum }Sa\left(u\right)p\left(u\right)$$

where the sum is with respect to all hidden variables *u*, which are present in our sequence of dendrograms. In the simplest situation,

*≪ Sa ≫≠<Sa>*

The deviation should be visible, not just a very small deviation.

### 10.2. Scheme for Determining if Correlations between Data Are Nonergodic

However, it can be that at the level of one sequence, the ergodicity holds, but it is violated for correlations (A. Khrennikov, one of the current authors, has a paper about this possibility and it seems that violation of CHSH can be of this type).

Then, we proceeded without the random choice and calculated for *A* = (*A*1, *…*, *An*), *B* = (*B*1, *..., bN*) *Sa and Sb as in the first method of Step 3 in Section 9*. Thus, our frequency correlation is as follows:

(5) $$<Sa,Sb>=(\overset{n}{\underset{j=1}{\sum }}Sa\left(A_{j}\right)Sb\left(B_{j}\right))/N$$

Then, we completed this analysis by the measure-theoretic framework. We considered all possible pairs of hidden variables, *d*-vectors, *u,v*, which are present in the pairs *Aj, Bj*, and found the probability number of occurrences of u**,v, as follows:

(6) $$p\left(u,v\right)=\frac{number\;of\;occurrences\;of\;u,v}{n}$$

and

(7) $$\ll Sa,Sb\gg =\underset{u}{\sum }Sa\left(u\right)Sb\left(v\right)p\left(u,v\right)$$

and for nonergodic correlations,

*<<Sa,Sb>>* ≠ *<Sa,Sb>*.

## 11. Results

We define the two distance metrics as follows:

Euclidean: $euclidean\;distance=\sqrt{{\left(x_{i}-x_{j}\right)}^{2}}$, where x∊spatial data point, i,j∊1,2…n. n=d block size.
Minkowski: $minkowski\;distance=-{\left(t_{i}-t_{j}\right)}^{2}+{\left(x_{i}-x_{j}\right)}^{2}$, where x∊spatial data point, i,j∊1,2…n.n=d block size, t∊temporal data point, i,j∊1,2…n. n=d block size.

We analyzed the same datasets by applying the Section 9 scheme (first and second methods) to the calculation of dendrogramic correlations for **three different “views”** of the datasets:

1. We constructed dendrograms with the Euclidean distance metric, which is shown in Section 1 of the results.
2. We constructed dendrograms with the Minkowski distance metric, which is shown in Section 2 of the results.
3. We Lorentz-transformed the datasets and then constructed dendrograms with the Minkowski distance metric.

### 11.1. CHSH Violations for 2-Slit Diffraction Experiment Data

As an example, we reproduced the Bell violations of correlations from a very classical double-slit diffraction experiment [73]. We refer to parts of the working flow process in Figure 1.

The original experiment used a CCD camera with a 512 × 512 detector chip. The detection pattern in each frame in the experiment was represented as a binary matrix of 512 × 512, where values of 0 represent no detection of photons in the corresponding detector, while values of 1 represent the detection of photons in the corresponding detector. For each frame and its corresponding binary matrix, we found all column positions that had the value 1, multiplied these positions’ values, and calculated the log10 value of the outcome; this resulted in unique numbers representing each frame’s detection pattern (Figure 1 steps A and B).

Thus, each frame was encoded with only one unique number A_f_ and time number T_f_, in which f ∈ frame 1, 2, …, N, N = 4070. From each fixed number of d consecutive frames, d = 4, 5, …, 9 frames, and the pairwise distances between each of the d A_f_ (or d vectors (T_f_ A_f_)) were calculated (Figure 1 step C).

The pairwise distances were calculated according to the above three “**views”** of the datasets: first, by applying the Euclidean distance metric between each of the A_f_ values, secondly, by applying the Minkowski distance metric between each of the two vectors *(T_f_ A_f_), and thirdly, by applying the Lorentz transformation to each vector (T_f_ A_f_), where the reference frame was 0.99 the speed of light, and then each Lorentzian-transformed vector [T_f_ A_f_]_lorentzian_ was compared to another Lorentzian-transformed vector by applying the Minkowski distance metric between such vectors. 

Dendrograms were constructed from the pairwise distances (Figure 1 step D) by using a ward or a weighted linkage algorithm. The result was a series of consecutive dendrograms, *D*1, *D*2, …, *Dn*, where n = ⌊N/d⌋ with the block length/edge number in a single dendrogram d = 4, 5, …, 9. For each such d, we split the dendrogram series into two series (the first chronological half and the second chronological half) of dendrograms, each with a length of n/2 (the above process is shown in Figure 1). One series was the observable Alice measures and the second series was the observable Bob measures. As in Step 2 of Section 9, we represented each dendrogram edge *Dn* with a natural number (as shown in Figure 1 step D). These features were sorted into ascending order according to the hierarchical structure of the dendrogram. We then evaluated the CHSH correlations in accordance with the first and second methods of Step 3.

### 11.2. CHSH First Method 

**Figure 2:**
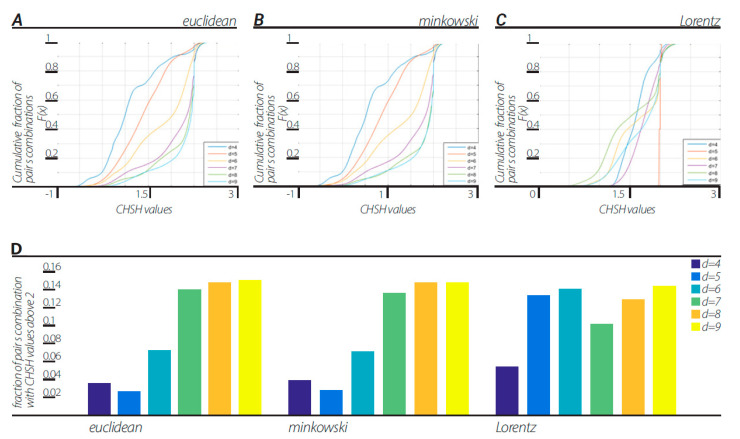
CHSH values computed according to the first method in Section 9 step 3. (**A**) Cdf’s of CHSH values with Euclidean view under “ward” linkage clustering algorithm. Each cdf corresponds to block size d = 4, 5, …, 9. (**B**) Cdf’s of CHSH values with Minkowski view under “ward” linkage clustering algorithm. Each cdf corresponds to block size d = 4, 5, …, 9. (**C**) Cdf’s of CHSH values with Lorentzian view under “ward” linkage clustering algorithm. Each cdf corresponds to block size d = 4, 5, …, 9. (**D**) fraction of CHSH with values above 2 in each view, Euclidean, Minkowski and Lorentzian, with block sizes d = 4, 5, …, 9.

In line with the first method of Step 3 in Section 9 (numerical example in Illustration 1E), we calculated the CHSH values for each combination of features *ab* = [ [*a b*] [*a b*’] [*a*’ *b*] [*a*’ *b*’]], where *a, a’, b*, and *b’* were chosen out of the number of features in the whole two-dendrogram series. Cumulative distribution functions (CDFs) of CHSH values of all pair combinations are shown in Figure 2. Dendrograms were constructed with block sizes of d = 4, 5, …, 9 in three different ways corresponding to the three views described above. All views in all block sizes of d = 4, 5, …, 9 showed a fraction of the pairs producing CHSH values above 2, resulting in a violation of CHSH inequality.

**Figure 3:**
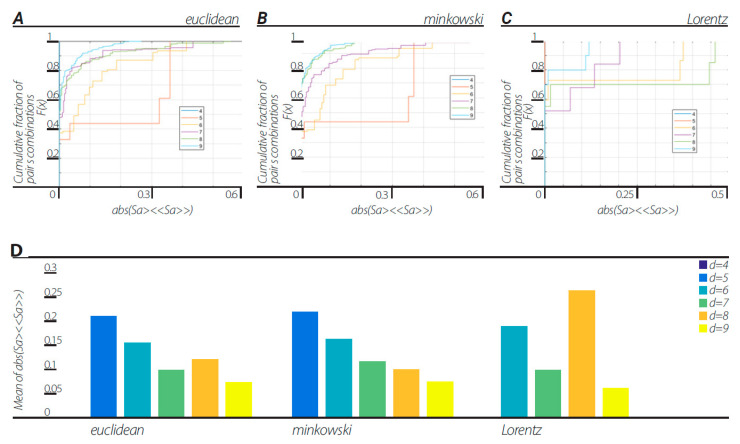
Ergodicity score values were computed according to the scheme described in Section 10.1. (**A**) CDFs of ergodicity score values with Euclidean view under “ward” linkage clustering algorithm. Each CDF corresponds to a block size of d = 4, 5, …, 9. (**B**) CDFs of ergodicity score values with Minkowski view under “ward” linkage clustering algorithm. Each CDF corresponds to a block size of d = 4, 5, …, 9. (**C**) CDFs of ergodicity score values with Lorentzian view under “ward” linkage clustering algorithm. Each CDF corresponds to a block size of d = 4, 5, …, 9. (**D**) Mean of ergodicity score in each view, Euclidean, Minkowski, and Lorentzian, with block sizes of d = 4, 5, …, 9.

In order to verify that nonergodicity is the cause of the violation of the CHSH inequality, we tested the amount of ergodicity of the dendrogramic data (Figure 3). With the same data that the CHSH values were calculated from, we tested the data for any pair of features a = [a1 a2], as described in Section 10.1. The results show that the dendrogramic data for dendrograms of block sizes d = 5, 6, …, 9, the mean ergodicity score (|<Sa>−<<Sa>>|) shows considerable fractions of all possible *a* = [*a1* *a*2] pairs with a non-zero value, indicating clear nonergodicity. Moreover, cross-correlating the fractions of CHSH values above 2 and the data ergodicity score in each dendrogram of block sizes d = 5, 6, …, 9 for the Euclidean metric view, Minkowski view and Lorentzian view resulted in correlation coefficients of −0.9485, −0.9772, and −0.0025 respectively.

**Figure 4:**
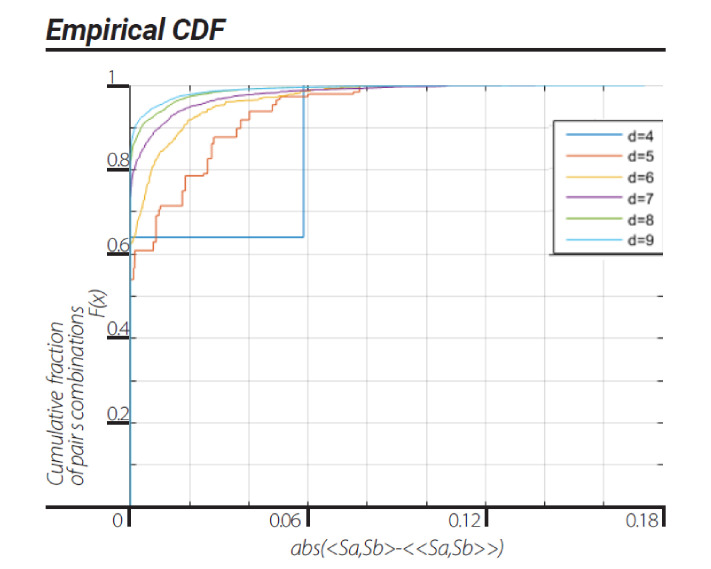
Ergodicity score values computed according to the scheme described in Section 10.2. CDFs of ergodicity score values with Euclidean view under “ward” linkage clustering algorithm. Each CDF corresponds to a block size of d = 4, 5, …, 9.

We note that for all views, the dendrogram data series with block sizes of d = 4 show that the data is ergodic, thus the violation of CHSH inequality for a block size of d = 4 can be a result of correlation nonergodicity. We carried on to investigate ergodicity in terms of two stationary ergodic series correlations in the Euclidean view. This ergodic test for correlations is described in Section 10.2. The results of this investigation clearly show all d = 4, 5, …, 9 block size dendrograms are nonergodic, in terms of correlations, in the Euclidean view (Figure 4).

### 11.3. CHSH Second Method 

In line with the second method of Step 3 in Section 9 (numerical example in Figure 1 step E), we calculated the CHSH values for each combination of features

**Figure 5:**
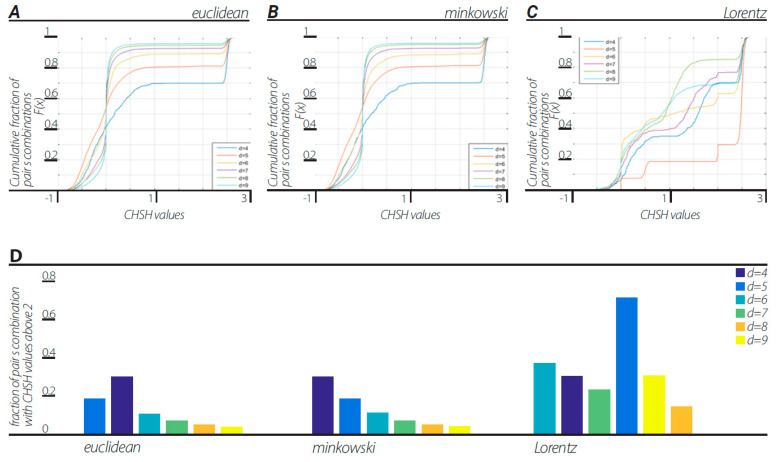
CHSH values computed according to the second method of Step 3 in Section 9. (**A**) CDFs of CHSH values with Euclidean view under “ward” linkage clustering algorithm. Each CDF corresponds to a block size of d = 4, 5, …, 9. (**B**) CDFs of CHSH values with Minkowski view under “ward” linkage clustering algorithm. Each CDF corresponds to a block size of d = 4, 5, …, 9. (**C**) CDFs of CHSH values with Lorentzian view under “ward” linkage clustering algorithm. Each CDF corresponds to a block size of d = 4, 5, …, 9. (**D**) Fraction of CHSH with values above a value of 2 in each view: Euclidean, Minkowski and Lorentzian, with block sizes d = 4, 5, …, 9.

*ab* = [[*a b*] [*a b*’] [*a*’ *b*] [*a*’ *b*’]]

where *a, a’, b*, and *b’* were chosen out of the number of features in the whole two-dendrogram series. Cumulative distribution functions (CDFs) of the CHSH values of all pair combinations are shown in Figure 4. Dendrograms were constructed with block sizes of d = 4, 5, …, 9 in three different ways corresponding to the three views described above. Again, as with the first method, all views in all block sizes of d = 4, 5, …, 9 show a fraction of the pairs producing CHSH values above 2, resulting in a violation of CHSH inequality (Figure 5). Interestingly, the fraction of CHSH values above 2 in the Euclidean and Minkowski views show descending fraction values in respect to increasing d values of d = 4, 5, …, 9 in contrast to the first method, which showed an ascending relation with increasing d values.

Please note that the Euclidean CDFs rise in correlation to the second ergodic test described in Figure 4.

**Figure 6:**
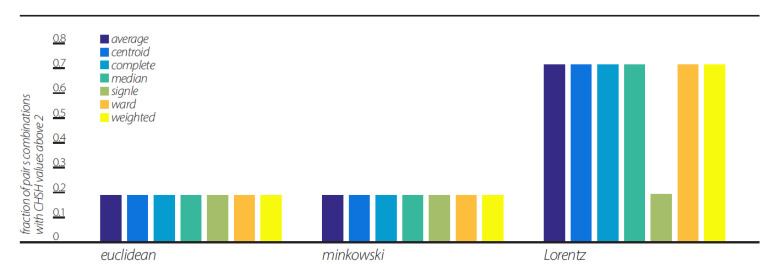
The influence of seven linkage clustering algorithms on CHSH inequality violation. Fractions of CHSH with values above 2 in each view, Euclidean, Minkowski, and Lorentzian, with block sizes of d = 5 and in each of the seven linkage clustering algorithms.

We further studied the dependence of the three views’ violations of CHSH inequality on the linkage clustering algorithm (Figure 6). For that purpose, we used seven different algorithms, all with d = 5, which showed no changes in the Euclidean and Minkowski views as to the fraction of pairs violating CHSH inequality. The Lorentz view showed a significant change in value in the “single” algorithm, resulting in fractions of pairs’ values comparable to the Euclidean and Minkowski views.

#### CHSH Second Method Random Data

We carried on to produce 10 random number sequences (comparable in size with the number of frames in the 2-slit diffraction experiment) in order to compare the analysis with the 2-slit diffraction experiment correlation analysis. 

For this purpose, for each random number in one of the ten sequences, we used the following scheme:

First, we randomly chose a number between 1 and 7 which indicated the number of random numbers generated from the interval [1, 512]; this was in correspondence with the 2-slit diffraction experiment with 512 detectors.

**Figure 7:**
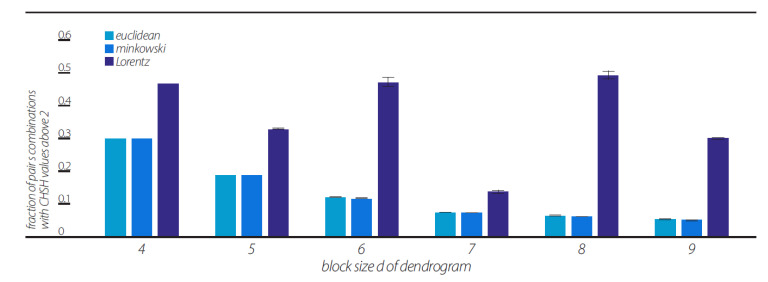
Mean CHSH values computed from 10 sequences of random data according to the second method of Step 3 in Section 9. Fractions of CHSH with values above 2 in each view, Euclidean, Minkowski, and Lorentzian, with block sizes of d = 4, 5, …, 9, and in each with a “ward” linkage clustering algorithm.

We multiplied these numbers and calculated the log10 value of the outcome, which resulted in a unique number representing each “randomly generated frame detection pattern”. Each such frame was accompanied by a timestamp, which indicated the time of generation of the “randomly generated frame detection pattern”. We then analyzed, in each of the “views” as defined in Section 10, the fraction of ab pairs that violated CHSH inequality. The mean fraction values of ab pairs of ten random sequences that violate the CHSH inequality are shown in Figure 7. 

### 11.4. Lorentz-Transformed Data

**Figure 8:**
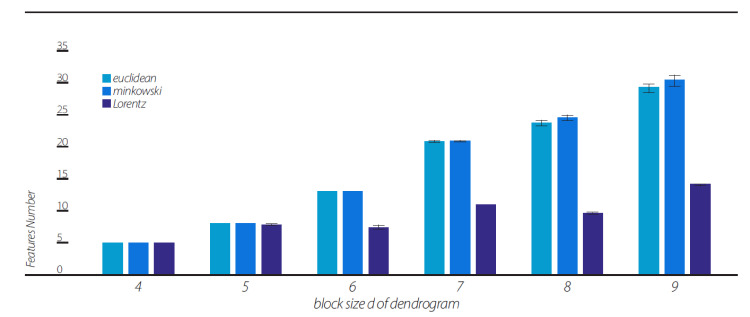
Mean feature numbers computed from 10 sequences of dendrograms composed out of random data. Mean feature number of 10 sequences in each view, the Euclidean, Minkowski, and Lorentzian and in block sizes of d = 4, 5, …, 9.

As was clearly indicated by the above analyses, the Lorentz-transformed data resulted in higher fractions of pairs violating the CHSH inequality under most of the clustering algorithms (although, the “single” algorithm showed comparable CHSH fraction values, see Figure 6). One might wonder why. The explanation for this phenomenon is that most clustering algorithms, except for the “single” algorithm, produce dendrogram series with much a smaller phase space of the possible topology of dendrograms. This is clearly seen in Figure 8, which shows the number of features contained in the dendrogram series of the 10 random data sequences under each of the three views. The number of features in the Lorentzian view remains small for all d = 4, 5, …, 9.

## 12. Discussion on the Results of Numerical Data Analysis

To emphasize our basic idea, quantum-likeness is not the property of systems but of a time series of observations. In the same way as randomness, quantum-likeness can be established on the basis of tests for data. For this study, we considered only one (but very important) test, the CHSH test. The following are our main results:

(a) Violation of the CHSH inequality can be demonstrated by a time series generated by measurements on classical systems.
(b) The key point is a hierarchic dendrogramic representation of data. It can be described by a local hidden variable model.
(c) CHSH violation is closely correlated with a violation of ergodicity.

Generally, nonergodicity is the fundamental property of a hierarchic dendrogramic representation of statistical data.

This representation is based on the application of clustering algorithms. Although different algorithms can generate different dendrogramic series from the same experimental data, general properties of representations do not differ; all algorithms show CHSH violation and nonergodicity of data or of correlations. Of course, the degrees of violation and nonergodicity vary with algorithms (see Figure 2, Figure 3, Figure 4 and Figure 5 and Figure 7).

By taking into account the temporal component of the data series, we generated two-dimensional geometry endowed with the Minkowski pseudo-metric. We applied DH theory to this geometry. It was found that the basic properties of the Minkowski DH theory do not differ from its Euclidean version (Figure 2, Figure 3, Figure 4 and Figure 5 and Figure 7). For the last step of our playing with algorithms, we performed a Lorentz transformation of data and then proceeded with dendrogramic modeling. Again, the Lorentz-transformed data showed CHSH violations and nonergodicity of data (Figure 2, Figure 3, Figure 4, Figure 5 and Figure 6), although not with comparable values to the Euclidean and Minkowski views. We show that these CHSH violations can become comparable with the violations under the Euclidean and Minkowski views under certain clustering algorithms (Figure 6, “single” clustering algorithm). We also show that the high fraction of pairs showing CHSH violations in the Lorentzian view is a consequence of the smaller phase space of the possible topology of dendrograms under most clustering algorithms, which result in a smaller number of features (Figure 8).

The two methods in Step 3 of Section 9 use the hierarchical information of the data differently. As a result, these two method adaptations to the formalization of the CHSH experiment, in terms of the hierarchical information, are different. This different adaptation leads to somewhat different results, where CHSH violations seem to be with the opposite phase with a d block size. Although such correlations disappeared under Lorentz transformation, we note that, as shown in Figure 2, we used a particular linkage algorithm that behaves differently for Lorentz-transformed data, as can be seen in all figures. Interestingly, Figure 6 shows that the “single” linkage method results in the same performance for the Euclidean, Minkowski, and Lorentz “views”. Thus, we suspect that these correlations will be recovered under the “single” linkage algorithm. Although this still needs to be investigated, we note that both methods show CHSH violations. 

Some more simulations should be considered with a d block size larger than 9 in order to again verify the transition from quantum to classical correlations already discussed in the previous study [1].

## 13. Concluding Remarks

In this study, DH theory [1] was structured in the ontic–epistemic (implicate–explicate order) framework. The experimental data was described by dendrograms generated by clustering algorithms—the epistemic counterpart of our theory. In the limit, dendrograms generated the infinite p-adic tree—the mathematical model of the ontic universe. 

DH theory is realistic, not only because the epistemic theory of observations has an ontic background but also because, even at the epistemic level, it is possible to introduce hidden variables. Ontic realism is very exotic; *the universe is zero-dimensional, totally disconnected, and disordered.* This universe is bounded, but with respect to the very special distance, the p-adic ultrametric. However, maybe it is even more surprising that the local realistic description can be used eventually at the epistemic level, with hierarchic hidden variables and observables as their functions. 

DH theory provides a novel viewpoint on classical–quantum interrelations. First, we stress that the ontic p-adic world is classical. In the epistemic dendrogramic world, simpler dendrograms have higher degrees of quantum-likeness. For the moment, the latter is characterized with just one test of quantumness—the CHSH test. Its violation is a consequence of the nonergodicity of empirical data. However, it is crucial that this data is represented dendrogramically. This representation gives us a local hidden variable model that generates correlations that violate the CHSH inequation (cf. [67,68,69,74,75]). The violation of ergodicity is closely coupled with the interrelation of the Kolmogorov measure-theoretic and the von Mises frequency approaches to probability (see [68] and references therein). 

Finally, we noticed that the size of the baselevel of a dendrogram can be considered as a system’s dimension. It is interesting that we are able to violate the CHSH inequality only for d > 3. 

## Figures and Tables

**Figure A1:**
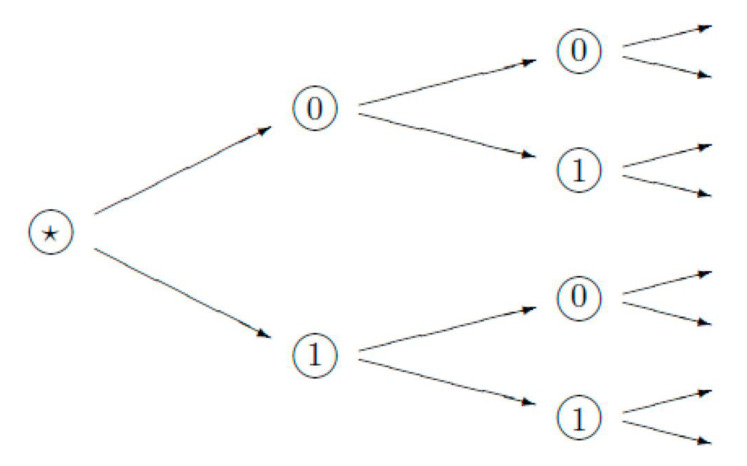
2-adic hierarchic tree.

## Data Availability

No experimental data was collected.

## Appendix A. Ultrametric Spaces, Trees, p-Adic Numbers I

In this appendix, we follow the non-expert friendly presentation from paper [76]. In some applications, the point structure of a set *X* and the properties of a metric ρ may essentially differ from the Euclidean case. We are interested in metric spaces *X* where, instead of the standard triangle inequality, the strong triangle inequality,

ρ (*x, y*) *≤ max*[ρ(*x, z*), ρ(*z, y*)] (A1)

is valid. Such a metric is called an ultrametric, and such metric spaces are called ultrametric spaces. The strong triangle inequality can be stated geometrically: all triangles are isosceles.

Let us discuss the main properties of ultrametric space *X.* We set

*B_r_*(*a*) *=* {*x* ∈ *X:* ρ*(x − a) < r*}*, B_r_* ~ (*a*) *=* {*x* ∈ *X:* ρ(*x − a*) *≤ r*}*, r > 0, a* ∈ *X* (A2)

These are balls of the radius *r* with the center at the point *a.* Our standard intuition tells us that *B_r_*(*a*) is a closed ball, but not open, and *B_r_* ~ (*a*) is an open ball, but not closed. However, it is not valid for ultrametric spaces.

In an ultrametric space, each ball in *X* is open and closed at the same time. Each point of a ball may serve as a center. A ball may have infinite radii.

Let *U* and *V* be two balls in ultrametric space *X.* Thus, there are only two possibilities: (1) the balls are ordered by inclusion (i.e., *U* ⊂ *V* or *V* ⊂ *U*); (2) the balls are disjointed.

Thus, if two balls have a common point, then one has to be a part of another.

The symbol *S_r_*(*a*) denotes the sphere {*x* ∈ *X:* ρ(*x, a*) *= r*} of the radius *r > 0* with the center at *a.* There is also a large deviation from the Euclidean case; the sphere *S_r_*(*a*) is not a boundary of *B_r_*(*a*) or *B_r_* ~ (*a*).

Consider the following class of ultrametric spaces (*X*, ρ). Every point x has an infinite number of coordinates *x =* (*a_o_, a*_1_*, ..., a_n_*). Each coordinate yields a finite number of values *a* ∈ {0*, ..., m −* 1}**, where *p >* 1 is a natural number. We denote the space of sequences (1) by the symbol *X* = **Z****p**. The standard ultrametric is introduced in this set in the following way.

Let *x* = (*a*_0_, *a*_1_, *a*_2_, ..., *a_n_*), *y* = (*b*_0_, *b*_1_, *b*_2_, ..., *b_n_*) ∈**Z****p**. We set

r_p (*x*, *y*) = l/*m^k   ^* if *a_j_* = *b_j_*, *j* = 0, 1, ..., *k* − 1, and *a_k_* ≠ *b_k_* (A3)

This is a metric and even an ultrametric. To find the distance r_p(*x, y*) between two strings of digits, *x* and *y*, we have to find the first position *k* at which the strings have different digits. The space *X =* Zm coincides with the unit ball centered at zero, *X = B*_1_(0); this space is compact. Geometrically, it can be represented by the tree (see Figure 8 for the 2-adic tree representing **Z****2**). Here, one vertex, the root labeled as *R*, is incidental for two edges and other vertices are incidental for three edges. We noticed that it is convenient to consider this tree as the directed graph; for each vertex *I* different from *R*, one edge comes from the branch starting at *R*, the “input edge”, and two edges go out from *I*, the “output edges”. These two edges (or vertices at their ends) are labeled by *a =* 0, 1. In Figure A1, the order of labeling of the output edges is based on the embedding of the tree in the plane, the upper output edges are labeled by 0 and the lower by 1. This leads to the concrete numerical representation of this tree. However, the rule used for the labeling of edges is not obligatory; for each vertex *I*, we can assign 0/1 to each of the output edges in an arbitrary way and obtain another numerical representation of this tree.
